# Cu_x_O-Modified Nanoporous Cu Foil as a Self-Supporting Electrode for Supercapacitor and Oxygen Evolution Reaction

**DOI:** 10.3390/nano12122121

**Published:** 2022-06-20

**Authors:** Zhenhan Li, Jianbin Lin, Xin He, Yue Xin, Ping Liang, Chi Zhang

**Affiliations:** School of Applied Physics and Materials, Wuyi University, 99 Yingbin Road, Jiangmen 529020, China; zhenhanli@yeah.net (Z.L.); wylinjianbin@163.com (J.L.); hexinwyu@126.com (X.H.); xin3231946@163.com (Y.X.); ping_liang@126.com (P.L.)

**Keywords:** nanoporous copper, dealloying, copper oxide, supercapacitor, oxygen evolution reaction

## Abstract

Designing and modifying nanoporous metal foils to make them suitable for supercapacitor and catalysis is significant but challenging. In this work, Cu_x_O nanoflakes have been successfully in situ grown on nanoporous Cu foil via a facile electrooxidation method. A Ga-assisted surface Ga-Cu alloying–dealloying is adopted to realize the formation of a nanoporous Cu layer on the flexible Cu foil. The following electrooxidation, at a constant potential, modifies the nanoporous Cu layer with Cu_x_O nanoflakes. The optimum Cu_x_O/Cu electrode (O-Cu-2h) delivers the maximum areal capacitance of 0.745 F cm^−2^ (410.27 F g^−1^) at 0.2 mA cm^−2^ and maintains 94.71% of the capacitance after 12,000 cycles. The supercapacitor consisted of the O-Cu-2h as the positive electrode and activated carbon as the negative electrode has an energy density of 24.20 Wh kg^−1^ and power density of 0.65 kW kg^−1^. The potential of using the electrode as oxygen evolution reaction catalysts is also investigated. The overpotential of O-Cu-2h at 10 mA cm^−2^ is 394 mV; however, the long-term stability still needs further improvement.

## 1. Introduction

Though dealloying has gained fruitful achievements during the past decades, the exploration of applying dealloyed materials in catalysts, sensors, and energy-storage devices never stops [1,2,3,4]. Dealloying is a top-down strategy that dissolves the active elements from an alloy and generates a nanostructure (mostly nanoporous structure) after the diffusion/reorganization of the less active elements [5,6]. Nanoporous noble metals, e.g., Au, Ag, and Cu, are among the most common materials created by dealloying. Nanoporous Au has been a prototype to study the mechanisms of dealloying, and it is widely applied as catalysts for fuel cells, methane pyrolysis, supercapacitors, actuator, and solar steam generation, etc. [4,7,8,9,10,11]. Nanoporous Cu has also been extensively studied, due to the relatively low price and great potential applications.

The study of dealloying Cu-based alloys can be dated back to the 1980s, when Cu-Mn alloys were dealloyed to probe into the morphology and composition of dealloyed products [12]. Raney^®^ Cu, produced by dealloying Al_2_Cu in a NaOH solution, is a successful catalyst for methanol production from synthesis gas [13]. Similar to Raney^®^ Cu, most of the dealloyed Cu materials are powdery. In addition to powdery nanoporous Cu, a self-supporting nanoporous Cu film is necessary for some application scenarios, e.g., electrodes of supercapacitors and lithium-ion batteries [14,15,16]. Nanoporous metal films can be fabricated through dealloying metallic glass ribbons, which are flexible due to the amorphous structure [17,18,19]. Another approach is to create an alloy layer on the metal film, followed by surface dealloying to generate a nanoporous layer. For instance, Diao et al. used a co-sputtering and dealloying method to fabricate bi-continuous and flexible nanoporous Cu films integrated onto the Cu foil [20]. In a previous study, the Ga-Cu alloy layer was created on a Cu foil via the mutual diffusion of Ga and Cu; a nanoporous Cu layer was generated after dealloying. The as-fabricated Cu foil was flexible with a nanoporous surface layer [21]. This method could be a facile and powerful technique to fabricate flexible nanoporous Cu foil for various applications. CuO or Cu_2_O oxide layers can easily form on the surface of nanosized Cu, which could act as catalysts or electrodes for supercapacitors [22,23,24,25,26,27,28]. Dong et al. applied an electrooxidation method to the Cu foam in an H_2_C_2_O_4_ solution to form a Cu_2_O/Cu electrode for the supercapacitors [27]. Li et al. fabricated a Cu_2_O/CuO nanosheet layer on nanoporous Cu via heat treatment [23]. 

Inspired by the above investigation, we fabricated a nanoporous Cu layer on the Cu foil via a Ga-Cu alloying and dealloying strategy in this work. Following the dealloying process, an electro-oxidized method was used to form a Cu_x_O layer on the nanoporous Cu surface. Through electrooxidation at a constant potential for different durations, Cu_x_O consisting of different Cu_2_O and CuO amounts were formed. The co-existence of Cu_2_O and CuO might benefit the electrochemical performance of the electrode. The self-supporting Cu_x_O/Cu was applied as a positive electrode in supercapacitors. The optimum electrode delivered a maximum areal capacitance of 0.745 F cm^−2^ (410.27 F g^−1^) at 0.2 mA cm^−2^ and maintained 94.71% of the capacitance after 12,000 cycles. We assembled an asymmetric supercapacitor using the Cu_x_O/Cu foil as the positive electrode and active carbon (AC) as the negative electrode. The supercapacitor device had an energy density of 24.20 Wh kg^−1^, corresponding to the power density of 0.65 kW kg^−1^. This study also used the as-fabricated electrodes as the oxygen evolution reaction catalysts. Although the OER performance was acceptable, more work would be necessary, in order to improve the long-term stability.

## 2. Experimental

### 2.1. Preparation of Cu_x_O/Cu Foils

The commercial Cu foils (50 μm thick, 99.9 wt.%, Kejing, Shenzhen, China) were cleaned with deionized water and alcohol before painting. A certain amount of Ga ingot (Macklin, 99.99 wt.%, Macklin, Shanghai, China) was placed on a hotplate at 50 °C to guarantee the liquid state. The cleaned Cu foils were fixed on another hotplate (maintained at 40 °C) during the painting process. Liquid Ga was brushed onto the Cu foil using a paintbrush, then annealed at 150 °C for 8 h in a vacuum oven to form Ga-Cu alloys via reaction–diffusion. The annealed foils were dealloyed in a 0.2 M HNO_3_ solution at 40 °C, until no bubbles emerged. The dealloyed foils were preserved in a vacuum tank at room temperature. The electrooxidation process was conducted using a two-electrode system containing a dealloyed Cu foil and graphite rod. The dealloyed foils were electro-oxidized in a 1 M KOH solution using a two-electrode system at 1 V for 15 min, 30 min, 1 h, 2 h, and 5 h, respectively, to form a copper oxide layer on the foil surface. The obtained samples were named O-Cu-Xm or O-Cu-Xh, where X referred to the electrooxidation time; ‘m’ is short for ‘minute’, and ‘h’ is short for ‘hour’. For instance, O-Cu-15m refers to the sample that was electro-oxidized for 15 min. 

### 2.2. Fabrication of Asymmetric Supercapacitor Devices

In the asymmetric supercapacitor device, the Cu_x_O/Cu foil was used as the positive electrode, and the active carbon (AC) powders deposited on the Ni foams acted as the negative electrode. The fabrication details of the negative electrode can be seen in ref. [27]. The positive and negative electrodes were positioned face-to-face and separated by cellulose paper in a 1 M KOH electrolyte.

### 2.3. Characterizations of Catalysts

The phase compositions of the samples were characterized by X-ray diffraction (XRD, X’pert Pro, Malvern Panalytical, Almelo, Netherlands). The microstructural features were investigated by a field emission scanning electron microscope (SEM, Zeiss Sigma 500, Birmingham, UK) and transmission electron microscope (TEM, JEM F200, JEOL, Tokyo, Japan). The surface status of the samples was analyzed by X-ray photoelectron spectroscopy (XPS, K-Alpha, Thermo Scientific, Loughborough, UK). Raman spectra analyses were performed on a HORIBA LabRAM HR Evolution (HORIBA, Kyoto, Japan), using a laser source of 488 nm. The concentration of Cu ions in the electrolyte after OER tests was determined via inductively coupled plasma-optical emission spectrometry (ICP-OES, Agilent 5110, Santa Clara, CA, USA).

### 2.4. Electrochemical Measurements

The electrochemical performance of the electrodes and devices was analyzed on an electrochemical workstation (CHI660E, Shanghai, China) in a 1 M KOH electrolyte. A Pt electrode, Ag/AgCl electrode, and as-synthesized electrodes were used as the counter, reference, and working electrodes, respectively, in a three-electrode system. The cyclic voltammetry (CV) was operated under various scan rates from 5 to 100 mV s^−1^, between 0−0.6 V vs. Ag/AgCl. The galvanic charging and discharging (GCD) was conducted under the current densities of 0.2 to 1 mA cm^−2^, between 0−0.5 V vs. Ag/AgCl. The electrochemical impedance spectroscopy (EIS) was obtained at open circuit potential in the frequency range from 0.01 Hz to 1 MHz with a 5 mV amplitude. The O-Cu-2h//AC asymmetric supercapacitor was tested via CV under various scan rates of 5, 10, 30, 50, 70, and 100 mV s^−1^, between 0.6−1.6 V, as well as GCD under current densities of 2, 3, 4, 5, 6 and 7 mA cm^−2^, between 0.6−1.6 V. The calculation details of areal capacitance, specific capacitance, and energy and power densities can be found in references [29,30]. To estimate the mass of Cu_x_O on the surface, we measured the weight of the dealloyed Cu foils before and after oxidation. To obtain accurate mass loading, at least three foils were weighted for each oxidation time. 

Linear sweep voltammetry (LSV) was used to verify the possibility of using the electro-oxidized foils as OER catalysts. LSV tests were carried out in an O_2_-saturated 1 M KOH electrolyte, with a scanning rate of 5 mV s^−1^ at the potential range of 0.1 to 1.1 V vs. RHE. All LSV curves were iR-compensated to exclude the influence of solution resistance. Electrochemical impedance spectroscopy (EIS) was conducted in the same electrolyte from 1 MHz to 0.01 Hz at 0.7 V vs. Hg/HgO, with an amplitude of 5 mV. All the potentials were converted to the reversible hydrogen electrode (RHE) reference, according to E (vs. RHE) = E (vs. Hg/HgO) + 0.0591pH + 0.098. 

## 3. Results and Discussion

### The Composition and Microstructure Analysis

**Figure 1:**
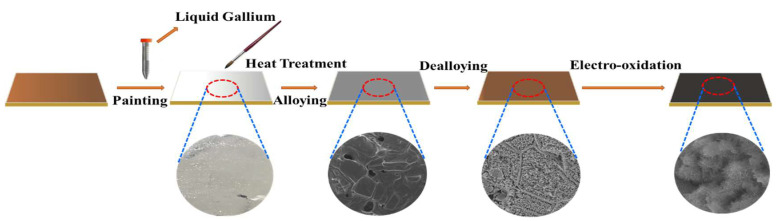
Schematic diagram of the fabrication process of the Cu_x_O/Cu electrodes.

**Figure 2:**
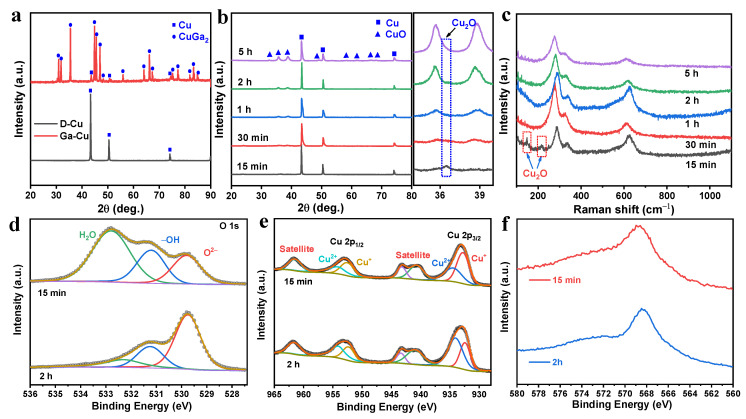
XRD patterns of (**a**) annealed Ga-Cu before and after dealloying and (**b**) dealloyed Cu film after being electro-oxidized for a different time; (**c**) Raman spectra; (**d**–**f**) XPS spectra of O 1s, Cu 2p, and Cu LMM for O-Cu-15m and O-Cu-2h.

A previous study reports that dealloying Ga-Cu alloy in an acidic solution resulted in the formation of nanoporous Cu [21]. In that report, the Ga-Cu alloy layer was formed via annealing at 100 °C for 5 h and generated a nanoporous structure after dealloying. Here, we fabricated a nanoporous structure on the surface of Cu film using the same strategy, followed by electrooxidation in an alkaline solution, the detailed procedure of which can be seen in Figure 1. After painting the Ga and annealing, the Ga_2_Cu phase (PDF #25-0275) formed on the surface of Cu foil (Figure 2a). The back-scattered SEM image shows the grain boundaries of the annealed Ga_2_Cu layer (Figure S1a). EDX elemental mapping shows the distribution of the Ga and Cu elements (Figure S1b–d). The EDX spectrum indicates that the Ga atomic concentration is 76.64 at.%, with that of Cu being 23.36 at.%, indicating that Ga was excessive on the surface after 8 h annealing at 150 °C (Figure S1e). The Ga_2_Cu layer can be fully dealloyed in a 0.2 M HNO_3_ solution with only Cu retained, as confirmed by the XRD pattern in Figure 2a. An electrooxidation method was applied to generate a Cu oxide layer on the dealloyed Cu surface. It has been reported that Cu_2_O nanoneedles could be grown on the etched Cu foam via CV cycling at –0.3–0.3 V vs. Ag/AgCl in the 1 M KOH solution [27]. Further oxidation at 0–0.6 V could transform Cu_2_O to CuO [31]. In another study, a Cu_2_O/CuO hybrid layer was formed on a nanoporous Cu ribbon by anodizing at the constant current densities in the 0.5 M KOH solution [23]. Inspired by the above method, we electro-oxidized the dealloyed Cu foil at a constant potential of 1 V in a two-electrode system, expecting to create a Cu_x_O layer on nanoporous Cu. Extra peaks are observed in the XRD patterns after electrooxidation for 15 min, 30 min, 1 h, 2 h, and 5 h. As shown in Figure 2b, a peak that appeared at 2θ 36.5° can be indexed to Cu_2_O (PDF #05-0667) [32]. With the electrooxidation time increasing to longer than 30 min, the relative intensity of the Cu_2_O peaks decreases. It shows almost only the CuO phase (PDF #48-1548), when the electrooxidation time is 5 h. The Raman spectra in Figure 2c demonstrate the presence of peaks at ~280, 330, and 620 cm^−1^, confirming the generation of CuO after electrooxidation [33,34]. Extra peaks can be found at 148 and 217 cm^−1^ for sample O-Cu-15m, ascribed to the characteristic Raman bands of Cu_2_O [35]. To further probe into the surface compositions of the Cu films, we conducted XPS on samples electro-oxidized for 15 min and 2 h. The XPS full surveys of O-Cu-15m and O-Cu-2h prove the existence of Cu and O (Figure S2). The O 1s spectra of both samples can be deconvoluted into three peaks at ~529.8, 531.2, and 532.3 eV, corresponding to lattice oxygen O^2−^, OH^−^, and adsorbed H_2_O on the surface (Figure 2d). From the deconvoluted results, the content of O^2−^ is calculated as 22.61 at.% for Cu-15min and 61.60 at.% for Cu-2h, indicating the generation of more CuO with extended oxidation duration. Cu 2p3/2 and Cu 2p1/2, along with their satellite peaks, are observed from the Cu 2p XPS spectra (Figure 2e). As for O-Cu-15m, a fitting peak located at 932.8 eV corresponds to Cu^+^, while the peak at 934.4 eV is indexed to Cu^2+^. Similar fitting results are observed in sample O-Cu-2h. The atomic ratio of Cu^+^ to Cu^2+^ is 1.49 for O-Cu-15m and 0.58 for O-Cu-2h, again indicating that more Cu_2_O exists in O-Cu-15m sample. The Cu LMM Auger spectra with the peak located at ~568.7 eV further prove the formation of Cu^2+^ (CuO) in both samples (Figure 2f). 

**Figure 3:**
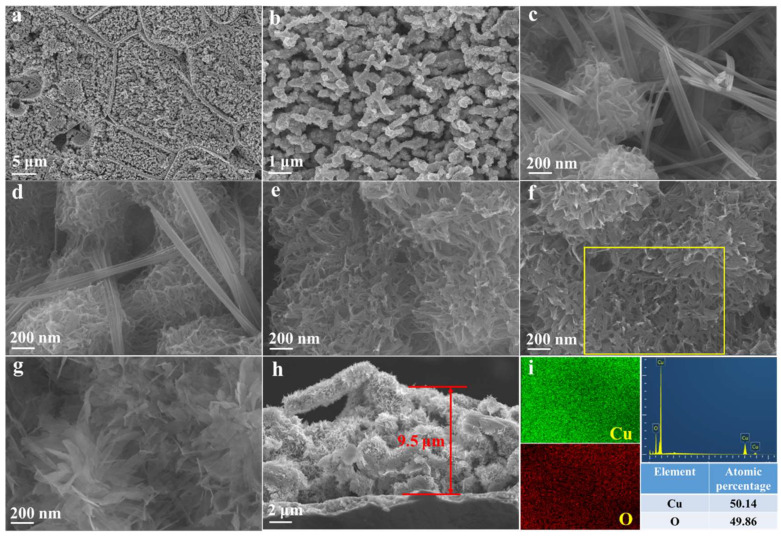
SEM surface view of (**a**,**b**) dealloyed Cu foil, (**c**–**h**) after electrooxidation for 15 min, 30 min, 1 h, 2 h, and 5 h, respectively; SEM cross-section view of (**h**) dealloyed Cu foil after electrooxidation for 2 h; (**i**) EDX mapping and spectrum corresponding the yellow rectangle area in (**f**).

After dealloying in 0.2 M HNO_3_ solution, nanoporous structures evolved on the Cu foil surface, as shown in Figure 3a,b. The grain boundaries can be observed on the surface of the dealloyed Cu foil, which inherits from the Ga-Gu alloys, as shown from the back-scattering SEM image (Figure S1a). The diameter of the dealloyed Cu ligament is ~200 nm, and no Ga reserves after dealloying (Figure S3). After electrooxidation in a 1 M KOH solution at 1.0 V vs. Ag/AgCl for 15 min, two distinct microstructures can be observed on the surface: nanobelts and nanoflakes. The EDX obtained from the rectangle area in Figure S4a,b shows the presence of Cu and O, with the atomic concentration of 34.29 and 65.71 at.%. The concentration ratio of Cu to O is 1.92, and it is approaching 2.0 for Cu_2_O. The EDX point scan from one typical nanoflake presents that the atomic ratio of Cu to O is 2.26; the atomic ratios from two typical nanobelts are 1.88 and 1.81 (Figure S4c). The EDX results further confirm the formation of Cu_2_O after electrooxidation for 15 min. With a prolonged oxidation length of 30 min, the microstructure (Figure 3d) is similar to O-Cu-15m. The atomic ratio of Cu to O from nanoflake and nanobelt areas are 1.50 and 1.33 (Figure S5), respectively, both smaller than that from O-Cu-15m. The microstructures of O-Cu-1h and O-Cu-2h are almost the same, while the nanoflakes seem thinner after 5 h oxidation (Figure 3e–g). At the initial oxidation stage, the nanoneedle Cu_2_O phase formed on the surface [27]. As the oxidation time increased, the nanoneedle or nanobelt became longer but less stable. Therefore, after 2 or 5 h oxidation, the nanobelts would grow into interlinked nanoflakes to make the structure more stable; the morphology can be observed in some CuO-based materials [23,36]. The atomic ratio of Cu to O from O-Cu-1h and O-Cu-2h keeps approximately 1, indicating that the dominated phase might be CuO (Figure 3i and Figure S6). After 5 h oxidation, the atomic ratio of Cu to O decreases to 0.73, which might suggest that the Cu was oxidized to higher valence after long-term oxidation (Figure S7). Moreover, a typical cross-section view of the oxidized foil displays that the oxidation layer is ~9.5 μm (Figure 3h). The cross-section view proves that the nanoflakes grow across the entire dealloyed layer. 

**Figure 4:**
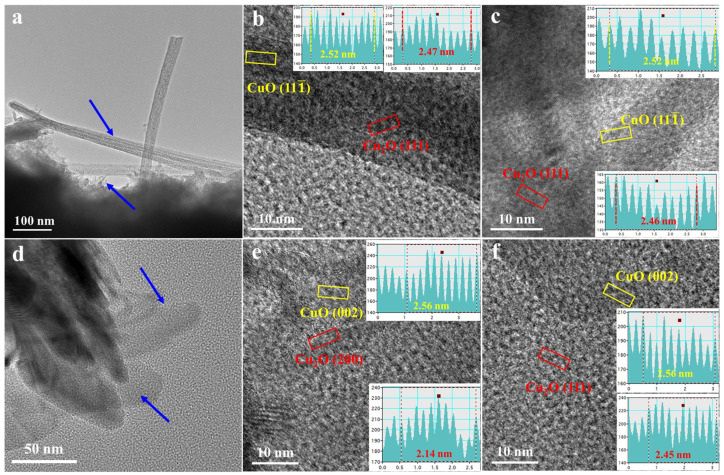
(**a**) TEM image of O-Cu-15m, (**b**) HRTEM image of a nanobelt area, (**c**) HRTEM image of a nanoflake area; (**d**) TEM image of O-Cu-2h, (**e**,**f**) HRTEM image of two typical nanoflake areas.

TEM and HRTEM were conducted to probe the microstructural features of O-Cu-15m and O-Cu-2h. Corresponding to the SEM results, nanobelts and nanoflakes are observed from O-Cu-15m (Figure 4a). The nanobelts are 10–30 nm in width. From the HRTEM result in Figure 4b, we observe two lattice distances, corresponding to (11$\bar{1}$) lattice plane from CuO and (111) lattice plane from Cu_2_O, respectively. The same lattice planes are also found in the nanoflakes (Figure 4c), indicating that both Cu_2_O and CuO generated during electrooxidation. For the O-Cu-2h sample, the lattice planes observed from one typical flake indicate the co-exitance of the CuO (002) and Cu_2_O (200) planes (Figure 4d,e). Another nanoflake shows the lattice plane from CuO (002) and Cu_2_O (111) (Figure 4f). The HRTEM analyses prove the formation of CuO and Cu_2_O after electrooxidation, which agrees with the XRD results. In a previous study, pure CuO could be fabricated by anodizing the dealloyed nanoporous Cu at 15 mA cm^−2^ and then calcining at 200 °C for 2 h [18]. This work also obtained the mixture of CuO and Cu_2_O after one-step electrooxidation at a constant potential. The XPS results prove that more CuO generated with a longer oxidation time. The electrode is nominated as Cu_x_O/Cu electrode in the following section.

**Figure 5:**
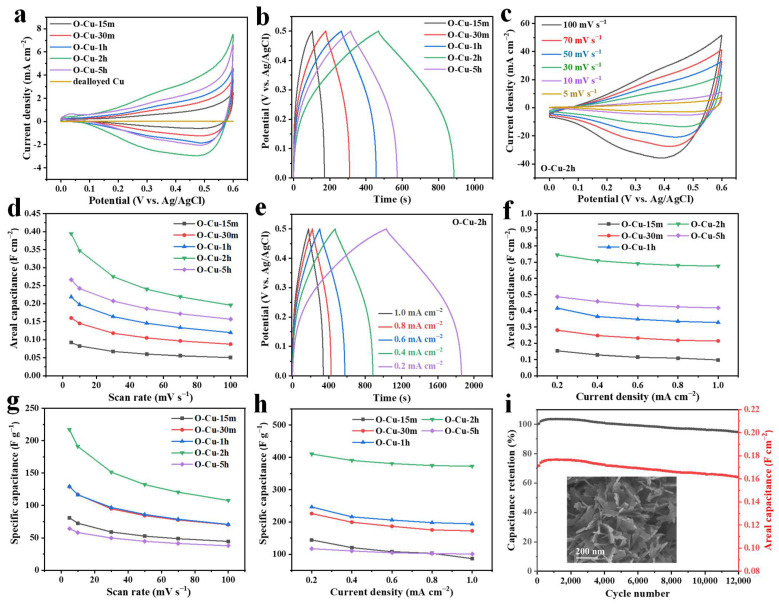
(**a**) Electrochemical performance of O-Cu-15m, O-Cu-30m, O-Cu-1h, O-Cu-2h, and O-Cu-5h in 1 M KOH. (**a**) CV curves at 5 mV s^−1^. (**b**) GCD profiles at 0.4 mA cm^−2^. (**c**) CV curves of O-Cu-2h at various scan rates ranging from 5 to 100 mV s^−1^. (**d**) Areal capacitance as a function of scan rate. (**e**) GCD profiles of O-Cu-2h at different current densities. (**f**) Areal capacitance versus the current density. (**g**) Specific capacitance normalized to the Cu_x_O mass as a function of scan rate. (**h**) Specific capacitance normalized to the Cu_x_O mass versus the current density. (**i**) Cyclic performance of O-Cu-2h measured at 100 mV s^−1^. The inset is the SEM image of O-Cu-2h after 12,000 cycles.

Cu_2_O or CuO is an excellent candidate for supercapacitors [37,38,39]. In this work, the Cu_x_O layer was grown on the flexible Cu foil, which could be potentially applied as a self-standing supercapacitor electrode. The performance of the as-synthesized Cu_x_O/Cu as a supercapacitor electrode was evaluated in a three-electrode cell containing a 1 M KOH electrolyte. Figure 5a compares the CVs of the oxidized and dealloyed samples recorded in the potential ranging from 0–0.6 V vs. Ag/AgCl at a 5 mV s^−1^ scan rate. The five electro-oxidized samples show almost the same CV curve shapes, indicating a similar reaction mechanism, which has been found in some other Cu_x_O-based electrodes [25,36]. The CV shape is different from those with an ideal rectangular shape generated from the electric double-layer capacitance [9,27]. O-Cu-2h has the largest integrated CV area, suggesting the most significant specific capacitance. In contrast, the pristine dealloyed Cu foil shows almost no capacitance at a 5 mV s^−1^ scan rate. Figure 5b displays the GCD curves of the samples oxidized for different times at a charge/discharge current density of 0.4 mA cm^−2^, and the curves also show a quasi-linear shape, suggesting the pseudocapacitive property [40]. It can be concluded from CV and GCD curves that O-Cu-2h has the maximum capacitance. Figure 5c presents the CV curves of O-Cu-2h recorded at scan rates ranging from 0–100 mV s^−1^, indicating a positive correlation between the integrated CV area and scan rate. The CV curves of the other four samples are displayed in Figure S8, which show a similar variation trend with O-Cu-2h. The areal capacitance calculated from the CV curves is exhibited in Figure 5d. The areal capacitance of O-Cu-2h can reach 0.394 F cm^−2^ at 5 mV s^−1^, and it decreases to 0.196 F cm^−2^ when the scan rate is 100 mV s^−1^. The areal capacitance values decrease in the sequence of O-Cu-2h, O-Cu-5h, O-Cu-1h, O-Cu-30m, and O-Cu-15m. At the current density of 0.2–1.0 mA cm^−2^, all the GCD curves have symmetrical triangle shapes, again indicating the pseudocapacitive property (Figure 5e). The GCD curves of the other four samples are displayed in Figure S9, showing the same charge/discharge features with O-Cu-2h. Figure 5f exhibits that O-Cu-2h has areal capacitance values of 0.745, 0.709, 0.691, 0.680, and 0.676 F cm^−2^ at 0.2 to 1.0 mA cm^−2^ current densities. The areal capacitance values calculated from GCD have the same performance sequence as those calculated from CV results. It is worth noting that O-Cu-5h has low capacitance, compared to O-Cu-2h. After oxidation for 5 h, almost all Cu_2_O transformed to CuO. Therefore, the pseudocapacitance that originated from the reversible reaction between Cu_2_O and CuO became less efficient. Moreover, the thicker oxide layer of O-Cu-5h may block the ionic transfer during the charge/discharge, resulting in lower capacitance. The mass loading of Cu_x_O was estimated via weighing the foil before and after electrooxidation, shown in Figure S10. It can be seen that the mass loading increased with longer electrooxidation times, namely increased from 1.13 to 1.82 of O-Cu-15m, and further to 4.15 mg cm^−2^ after 5 h oxidation. It should be mentioned that after 5 h oxidation, the oxide layer tends to be exfoliated from the foil during the operation. The specific capacitance normalized to the mass of Cu_x_O versus the scan rate, and current density is shown in Figure 5g,h, respectively. For instance, the sample O-Cu-2h presents the highest specific capacitance, possessing 410.27 F g^−1^ at 0.2 mA cm^−2^, much higher than 144.31, 226.01, and 246.42 F g^−1^ for O-Cu-15m, O-Cu-30m, and O-Cu-1h, respectively. O-Cu-5h has the lowest specific capacitance (117.30 F g^−1^ at 0.2 mA cm^−2^), due to the largest Cu_x_O mass on the surface. EIS was used to compare the conductivity and electrochemical dynamics of the electrode under different oxidation duration. Taking O-Cu-2h and O-Cu-15m as examples, the Nyquist plots of the two samples obtained in the frequency range of 1 MHz to 0.01 Hz are displayed in Figure S11. The internal resistance of O-Cu-15m is smaller than that of O-Cu-2h, indicating the thicker oxide layer with a longer oxidation duration. In the low frequency region, the steeper slope of O-Cu-2h reveals faster ionic diffusion between the oxide layer and electrolyte. The cycling stability of O-Cu-2h was evaluated via CV cycling at 100 mV s^−1^ for 12,000 cycles. The areal capacitance decreased from the initial value of 0.171 to 0.162 F cm^−2^ after cycling, retaining 94.71% of the initial capacitance (Figure 5i). The electrode has unchanged phase compositions after cycling, and the Cu_x_O flakes become thicker than the initial structure (Figure S12 and inset of Figure 5i). We compared the specific capacitance of Cu_x_O-based electrodes, as shown in Table S1 [25,26,27,36,38,39,41,42,43,44,45], and the results reveal that our electrode is comparable to the pure Cu_x_O material but inferior, compared to the Cu_x_O-based composites. Further investigations might be carried out to fabricate hybrid electrodes based on our materials.

**Figure 6:**
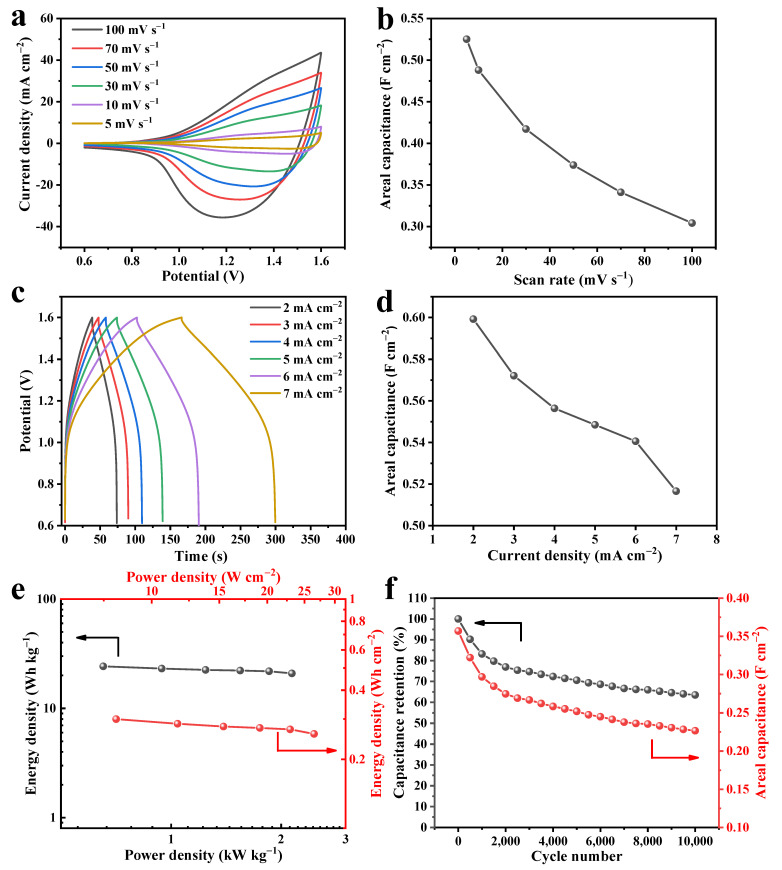
Electrochemical performance of the O-Cu-2h//AC asymmetric supercapacitor in the 1 M KOH solution. (**a**) CV curves at various scan rates. (**b**) Areal capacitance as a function of scan rate. (**c**) GCD curves at different current densities. (**d**) Areal capacitance as a function of current density. (**e**) Ragone plots of the O-Cu-2h//AC asymmetric supercapacitor. (**f**) Stability of the device for 10 k cycles.

The electrochemical properties of AC under various scan rates and current densities are present in Figure S13. The CV curves of the AC electrode show no redox peaks, and the symmetrical GCD curves have the typical characteristics of electric double-layer capacitance [27]. The asymmetric supercapacitor constructed with O-Cu-2h foil and AC decorated Ni foam was investigated, in order to prove its potential in energy storage devices. Figure 6a presents the supercapacitor’s CV curves at scan rates ranging from 5 to 100 mV s^−1^ and a potential window of 0.6–1.6 V. The areal capacitance calculated from the CV curves is shown in Figure 6b. The areal capacitance reaches 0.53 F cm^−2^ at the 5 mV s^−1^ scan rate and decreases to 0.30 F cm^−2^ with a 100 mV s^−1^ scan rate. GCD curves in Figure 6c show an IR drop at the initial state of the discharging stage, which is an indicator of the internal resistance of the device [46]. The GCD curves present a non-linear feature, suggesting the faradaic process [40]. The areal capacitance under different current densities is calculated and shown in Figure 6d. The maximum areal capacitance is obtained at 2 mA cm^−2^, reaching 0.60 F cm^−2^. The areal capacitance decreases to 0.52 F cm^−2^ when the current density increases to 7 mA cm^−2^. The energy and power densities are essential for evaluating the performance of an energy storage device. Figure 6e shows the Ragone plots (areal and mass) of the asymmetric supercapacitor calculated from the GCD curves. The energy densities of our device are in the range of 20.86 to 24.20 Wh kg^−1^ (0.26 to 0.30 Wh cm^−2^), corresponding to the power densities of 2.14 to 0.65 kW kg^−1^ (26.49 to 8.08 W cm^−2^). The energy densities are competitive with some other Cu_x_O-based supercapacitors (Table S2) [26,27,36,38,39,43,44,45]. For instance, a 3D Cu_2_O@Cu nanoneedle arrays electrode had an energy density of 26.0 Wh kg^−1^ at power density of 1.8 kW kg^−1^ [27]. An all-solid-state supercapacitor using 3D nanostructured Cu_x_O-modified Cu foam, as an electrode showed an energy density of 25 μWh cm^−2^ when the power density was 3 mW cm^−2^ [36]. An all-solid-state asymmetric supercapacitor with Cu_x_O NWs NWs@CoS_2_, as the electrode delivered an energy density of 49.8 Wh kg^−1^ at a power density of 0.226 kW kg^−1^ [47]. Unfortunately, the areal capacitance decreased gradually from 0.357 to 0.227 F cm^−2^, only retaining 63.59% of the initial capacitance after 10,000 cycles at 100 mV s^−1^ (Figure 6f). More modifications of the Cu_x_O/Cu foil should be explored, in order to improve the stability of the device. 

**Figure 7:**
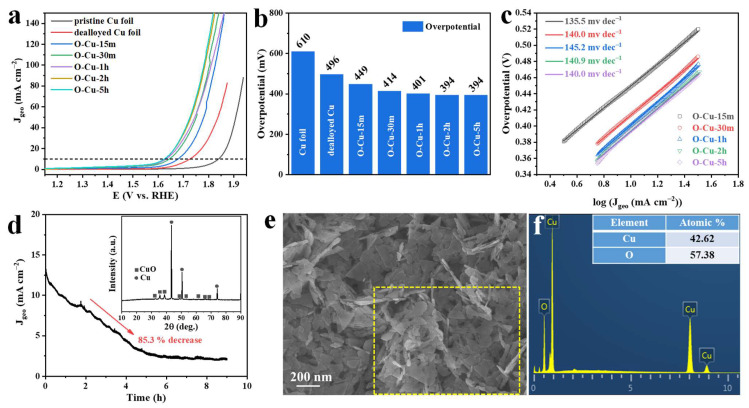
The OER performance of the oxidized samples. (**a**) LSV curves of pristine Cu foil, dealloyed Cu foil, O-Cu-15m, O-Cu-30m, O-Cu-1h, O-Cu-2h, and O-Cu-5h. (**b**) Overpotential obtained at 10 mA cm^−2^ current density. (**c**) Tafel slopes of the five oxidized samples. (**d**) i-t curves of O-Cu-2h at 1.65 V vs. RHE. (**e**) SEM image and (**f**) EDX spectrum of O-Cu-2h after stability test. The inset of Figure 6d is the XRD pattern of O-Cu-2h after the stability test.

There have been reports that CuO nanosheet bundles could be applied as efficient oxygen evolution reaction (OER) electrocatalysts [48]. In this study, we also investigate the OER property of the as-prepared samples. LSV curves of the pristine Cu foil, dealloyed Cu foil, and five oxidized samples are shown in Figure 7a. It can be seen that O-Cu-30m, O-Cu-1h, O-Cu-2h, and O-Cu-5h have comparable OER performance. The overpotential of O-Cu-2h and O-Cu-5h at 10 mA cm^−2^ is 394 mV, slightly lower than 401 and 414 mV of O-Cu-1h and O-Cu-30m, respectively. O-Cu-15m has the largest overpotential of 449 mV, smaller than dealloyed Cu and pristine Cu foil (Figure 7b). The overpotential is higher than the reported benchmark RuO_2_ (388 mV) [49]. The Tafel slope of all samples was around 140 mV dec^−1^, indicating the inferior catalytic kinetics (Figure 7c). Moreover, a Tafel slope higher than 120 mV dec^−1^ might indicate that the first step (M + OH^−^ → MOH) of OER in an alkaline solution is the rate-determining step [50]. The long-term stability was evaluated via a potentiostat test. The i-t curve in Figure 7d shows a rapid decline in the current density from the very beginning of the test, with an 85.3% decrease before it reaches a steady state. Although the phase compositions showed almost no change after the stability test (inset of Figure 7d), the surface Cu_x_O aggregated together to form thick flakes (Figure 7e). Moreover, the atomic ratio of Cu to O became 42.62 to 57.38%, appropriate to that of O-Cu-5h, indicating the continuous oxidation of the surface (Figure 7f). We noticed that the electrolyte gradually became light blue during the test. In order to observe the dissolution of Cu ions into the electrolyte, we also used a Pt plate counter electrode to conduct the i-t test. After the test, the surface of the Pt electrode clearly showed blue deposition, which could be deduced to Cu species (Figure S14). The ICP result confirmed 8.23 mg L^−1^ Cu ions in the electrolyte after the 9 h long-term test (Figure S14). In a previous study, a CuO nanowire@Co_3_O_4_ composite showed an overpotential of 258 mV at a current density of 10 mA cm^−2^ and Tafel slope of 72 mV dec^−1^ [51]. A 2D CuO on a stainless-steel substrate had an overpotential of 350 mV, with a Tafel slope of 59 mV dec^−1^ [48]. However, our electrode presented inferior OER performance, proving that the current as-prepared Cu_x_O/Cu electrode is unsuitable for OER application. In some previous studies, CuO was applied as an OER electrocatalyst when embedded or grown on other supporting materials, e.g., Co_3_O_4_ and graphene networks [42,51]. In the further study of the Cu_x_O/Cu materials in this study, the exploration of constructing some Cu_x_O-based hybrid might promote its application in OER. 

## 4. Conclusions

In summary, a Cu_x_O/Cu foil was developed via a Ga-Cu surface alloying–dealloying–electrooxidation strategy. The alloying process generated Ga2Cu alloys on the surface, and the following dealloying resulted in the formation of a nanoporous structure with a ligament size of ~200 nm. Cu_x_O flakes were grown on the Cu ligament, with the assistance of electrooxidation. The O-Cu-2h electrode had the areal capacitance of 0.745 F cm^−2^ at 0.2 mA cm^−2^, and 94.71% of the capacitance could be retained after 12,000 CV cycles. The O-Cu-2h//AC asymmetric supercapacitor delivered an energy density of 24.20 Wh kg^−1^, with a power density of 0.65 kW kg^−1^. Though the OER stability was inferior, further investigation could be explored, in order to improve the possibility of using the electrode as OER catalysts.

## Data Availability

Data presented in this article are available at request from the corresponding author.

## Supplementary Materials

The following supporting information can be downloaded at: https://www.mdpi.com/article/10.3390/nano12122121/s1, Figure S1: The SEM images of Ga-Cu surface. Figure S2: XPS full survey O-Cu-15m and O-Cu-2h. Figure S3: A typical EDX spectrum of the dealloyed Ga_2_Cu film corresponding to Figure 2b. Figure S4: SEM and EDS results of O-Cu-15m. Figure S5: SEM and EDS results of O-Cu-30m. Figure S6: SEM and EDS results of O-Cu-1h. Figure S7: SEM and EDS results of O-Cu-5h. Figure S8: CV curves of O-Cu-15m, O-Cu-30m, O-Cu-1h and O-Cu-5h at various scan rates. Figure S9: GCD curves of O-Cu-15m, O-Cu-30m, O-Cu-1h and O-Cu-5h at various current densities. Figure S10: The mass loading per unit area of Cu_x_O on the O-Cu-15m, O-Cu-30m, O-Cu-1h, O-Cu-2h, and O-Cu-5h. Figure S11: Nyquist impedance plots of O-Cu-2h and O-Cu-15m. Figure S12: XRD pattern and surface SEM image of O-Cu-2h after 12,000 CV cycles at 100 mV s^−1^. Figure S13: CV and GCD curves of AC. Figure S14: The comparison photos of Pt electrode before and after i-t test and the ICP result of the electrolyte after i-t test. Table S1: Comparison of Cu_x_O-based electrode for supercapacitors. Table S2: Comparison of the other asymmetric supercapacitors and this work of energy density and power density.

## References

[1] Wang Z., Liu J., Qin C., Yu H., Xia X., Wang C., Zhang Y., Hu Q., Zhao W. (2015). Dealloying of Cu-based metallic glasses in acidic solutions: Products and energy storage applications. Nanomaterials.

[2] McCue I., Benn E., Gaskey B., Erlebacher J. (2016). Dealloying and dealloyed materials. Annu. Rev. Mater. Res..

[3] Wu X., He G., Ding Y. (2020). Dealloyed nanoporous materials for rechargeable lithium batteries. Electrochem. Energy Rev..

[4] Zhang Y., Wang Y., Yu B., Yin K., Zhang Z. (2022). Hierarchically structured black gold film with ultrahigh porosity for solar steam generation. Adv. Mater..

[5] Erlebacher J., Aziz M.J., Karma A., Dimitrov N., Sieradzki K. (2001). Evolution of nanoporosity in dealloying. Nature.

[6] Erlebacher J. (2004). An atomistic description of dealloying. J. Electrochem. Soc..

[7] Li J., Yin H.-M., Li X.-B., Okunishi E., Shen Y.-L., He J., Tang Z.-K., Wang W.-X., Yücelen E., Li C. (2017). Surface evolution of a Pt–Pd–Au electrocatalyst for stable oxygen reduction. Nat. Energy.

[8] Xi W., Wang K., Shen Y., Ge M., Deng Z., Zhao Y., Cao Q., Ding Y., Hu G., Luo J. (2020). Dynamic co-catalysis of Au single atoms and nanoporous Au for methane pyrolysis. Nat. Commun..

[9] Lang X., Hirata A., Fujita T., Chen M. (2011). Nanoporous metal/oxide hybrid electrodes for electrochemical supercapacitors. Nat. Nanotechnol..

[10] Jin H.J., Wang X.L., Parida S., Wang K., Seo M., Weissmüller J. (2010). Nanoporous Au-Pt alloys as large strain electrochemical actuators. Nano Lett..

[11] Jin H.-J., Weissmüller J. (2011). A material with electrically tunable strength and flow stress. Science.

[12] Keir D., Pryor M. (1980). The dealloying of copper-manganese alloys. J. Electrochem. Soc..

[13] Smith A., Tran T., Wainwright M. (1999). Kinetics and mechanism of the preparation of Raney^®^ copper. J. Appl. Electrochem..

[14] Liu D., Yang Z., Wang P., Li F., Wang D., He D. (2013). Preparation of 3D nanoporous copper-supported cuprous oxide for high-performance lithium ion battery anodes. Nanoscale.

[15] Liu W., Zhang S., Li N., An S., Zheng J. (2013). Preparation and characterization of sandwich-typed three-dimensional nanoporous copper-supported tin thin-film anode for lithium ion battery. Int. J. Electrochem. Sci..

[16] Han G., Um J.H., Park H., Hong K., Yoon W.-S., Choe H. (2019). Hierarchically structured nanoporous copper for use as lithium-ion battery anode. Scr. Mater..

[17] Qin C., Zheng D., Hu Q., Zhang X., Wang Z., Li Y., Zhu J., Ou J.Z., Yang C., Wang Y. (2020). Flexible integrated metallic glass-based sandwich electrodes for high-performance wearable all-solid-state supercapacitors. Appl. Mater. Today.

[18] Zhang Q., Li M., Wang Z., Qin C., Zhang M., Li Y. (2020). Porous Cu_x_O/Ag_2_O (x = 1, 2) nanowires anodized on nanoporous Cu-Ag bimetal network as a self-supported flexible electrode for glucose sensing. Appl. Surf. Sci..

[19] Li R., Liu X., Wang H., Wu Y., Lu Z.P. (2016). Bendable nanoporous copper thin films with tunable thickness and pore features. Corros. Sci..

[20] Diao F., Xiao X., Luo B., Sun H., Ding F., Ci L., Si P. (2018). Two-step fabrication of nanoporous copper films with tunable morphology for SERS application. Appl. Surf. Sci..

[21] Wang Z., Wang Y., Gao H., Niu J., Zhang J., Peng Z., Zhang Z. (2018). ‘Painting’ nanostructured metals—Playing with liquid metal. Nanoscale Horiz..

[22] Kou T., Wang Y., Zhang C., Sun J., Zhang Z. (2013). Adsorption behavior of methyl orange onto nanoporous core-shell Cu@Cu_2_O nanocomposite. Chem. Eng. J..

[23] Li M., Li Y., Zhang Q., Qin C., Zhao W., Wang Z., Inoue A. (2019). Ultrafine Cu_2_O/CuO nanosheet arrays integrated with NPC/BMG composite rod for photocatalytic degradation. Appl. Surf. Sci..

[24] Liu W., Chen L., Cui L., Yan J., Zhang S., Shi S. (2019). Freestanding 3D nanoporous Cu@1D Cu_2_O nanowire heterostructures: From a facile one-step protocol to robust application in Li storage. J. Mater. Chem. A.

[25] Wang S., Jiang L., Hu J., Wang Q., Zhan S., Lu Y. (2020). Dual-functional Cu_x_O/Cu electrodes for supercapacitors and non-enzymatic glucose sensors fabricated by femtosecond laser enhanced thermal oxidation. J. Alloys Compd..

[26] Wu T., Xu L.N., Sun H., Bao Y., Yu H., Guo X., Hu Q., Li J. (2021). Hierarchical shell/core electrodes with CuO nanowires based on carbon cloths for high performance asymmetric supercapacitors. Ceram. Int..

[27] Dong C., Wang Y., Xu J., Cheng G., Yang W., Kou T., Zhang Z., Ding Y. (2014). 3D binder-free Cu_2_O@Cu nanoneedle arrays for high-performance asymmetric supercapacitors. J. Mater. Chem. A.

[28] Li Y., Zhao X., Liu H., Li W., Wang X. (2019). Synthesis and morphology control of nanoporous Cu_2_O/Cu and their application as electrode materials for capacitors. Nanomaterials.

[29] Liu J., Shen G., Zhao S., He X., Zhang C., Jiang T., Jiang J., Chen B. (2019). A one-dimensional Ag NW@NiCo/NiCo(OH)_2_ core–shell nanostructured electrode for a flexible and transparent asymmetric supercapacitor. J. Mater. Chem. A.

[30] Zhong Y., Chen B., Liang J., Xin Y., Zhang C., Zhang B., Wang S., He X. (2022). Structural engineering of ultrathin, lightweight, and bendable electrodes based on a nanowire network current collector enables flexible energy-storage devices. ACS Appl. Energy Mater..

[31] Yang W., Wang J., Ma W., Dong C., Cheng G., Zhang Z. (2016). Free-standing CuO nanoflake arrays coated Cu foam for advanced lithium ion battery anodes. J. Power Sources.

[32] Li M., Wang Z., Zhang Q., Qin C., Inoue A., Guo W. (2020). Formation and evolution of ultrathin Cu_2_O nanowires on NPC ribbon by anodizing for photocatalytic degradation. Appl. Surf. Sci..

[33] Xu J.F., Ji W., Shen Z.X., Li W.S., Tang S.H., Ye X.R., Jia D.Z., Xin X.Q. (1999). Raman spectra of CuO nanocrystals. J. Raman Spectrosc..

[34] Deng Y., Handoko A.D., Du Y., Xi S., Yeo B.S. (2016). In situ Raman spectroscopy of copper and copper oxide surfaces during electrochemical oxygen evolution reaction: Identification of cuIII oxides as catalytically active species. ACS Catal..

[35] Singhal A., Pai M.R., Rao R., Pillai K.T., Lieberwirth I., Tyagi A.K. (2013). Copper(I) oxide nanocrystals–one step synthesis, characterization, formation mechanism, and photocatalytic properties. Eur. J. Inorg. Chem..

[36] Li Y., Jiang H., Yan X., Zhu Y., Zhang W., Zhang M., Zhu W., Javed M.S., Pan J., Hussain S. (2021). 3D nanostructured Cu_x_O modified copper foam as a binder-free electrode for all-solid-state supercapacitor. Ceram. Int..

[37] Zheng M., Xiao X., Li L., Gu P., Dai X., Tang H., Hu Q., Xue H., Pang H. (2017). Hierarchically nanostructured transition metal oxides for supercapacitors. Sci. China Mater..

[38] Guo Y., Hong X., Wang Y., Li Q., Meng J., Dai R., Liu X., He L., Mai L. (2019). Multicomponent hierarchical Cu-doped NiCo-LDH/CuO double arrays for ultralong-life hybrid fiber supercapacitor. Adv. Funct. Mater..

[39] Moosavifard S.E., El-Kady M.F., Rahmanifar M.S., Kaner R.B., Mousavi M.F. (2015). Designing 3D highly ordered nanoporous CuO electrodes for high-performance asymmetric supercapacitors. ACS Appl. Mater. Interfaces.

[40] Jiang Y., Liu J. (2019). Definitions of pseudocapacitive materials: A brief review. Energy Environ. Mater..

[41] Wang K., Dong X., Zhao C., Qian X., Xu Y. (2015). Facile synthesis of Cu_2_O/CuO/RGO nanocomposite and its superior cyclability in supercapacitor. Electrochim. Acta.

[42] Li Y., Wang X., Yang Q., Javed M.S., Liu Q., Xu W., Hu C., Wei D. (2017). Ultra-fine CuO nanoparticles embedded in three-dimensional graphene network nano-structure for high-performance flexible supercapacitors. Electrochim. Acta.

[43] Chhetri K., Dahal B., Mukhiya T., Tiwari A.P., Muthurasu A., Kim T., Kim H., Kim H.Y. (2021). Integrated hybrid of graphitic carbon-encapsulated Cu_x_O on multilayered mesoporous carbon from copper MOFs and polyaniline for asymmetric supercapacitor and oxygen reduction reactions. Carbon.

[44] Kamble G.P., Rasal A.S., Gaikwad S.B., Gurav V.S., Chang J.-Y., Kolekar S.S., Ling Y.-C., Ghule A.V. (2021). CuCo_2_O_4_ Nanorods Coated with CuO Nanoneedles for Supercapacitor Applications. ACS Appl. Nano Mater..

[45] Zhan Y., Bai J., Guo F., Zhou H., Shu R., Yu Y., Qian L. (2021). Facile synthesis of biomass-derived porous carbons incorporated with CuO nanoparticles as promising electrode materials for high-performance supercapacitor applications. J. Alloys Compd..

[46] Stoller M.D., Ruoff R.S. (2010). Best practice methods for determining an electrode material’s performance for ultracapacitors. Energy Environ. Sci..

[47] Prasad Ojha G., Muthurasu A., Prasad Tiwari A., Pant B., Chhetri K., Mukhiya T., Dahal B., Lee M., Park M., Kim H.-Y. (2020). Vapor solid phase grown hierarchical Cu_x_O NWs integrated MOFs-derived CoS_2_ electrode for high-performance asymmetric supercapacitors and the oxygen evolution reaction. Chem. Eng. J..

[48] Pawar S.M., Pawar B.S., Hou B., Kim J., Aqueel Ahmed A.T., Chavan H.S., Jo Y., Cho S., Inamdar A.I., Gunjakar J.L. (2017). Self-assembled two-dimensional copper oxide nanosheet bundles as an efficient oxygen evolution reaction (OER) electrocatalyst for water splitting applications. J. Mater. Chem. A.

[49] Zhang S.L., Guan B.Y., Lu X.F., Xi S., Du Y., Lou X.W.D. (2020). Metal atom-doped Co_3_O_4_ hierarchical nanoplates for electrocatalytic oxygen evolution. Adv. Mater..

[50] Suen N.-T., Hung S.-F., Quan Q., Zhang N., Xu Y.-J., Chen H.M. (2017). Electrocatalysis for the oxygen evolution reaction: Recent development and future perspectives. Chem. Soc. Rev..

[51] Li X., Du X., Ma X., Wang Z., Hao X., Abudula A., Yoshida A., Guan G. (2017). CuO nanowire@Co_3_O_4_ ultrathin nanosheet core-shell arrays: An effective catalyst for oxygen evolution reaction. Electrochim. Acta.

