# Supplementary material for: CuxO-Modified Nanoporous Cu Foil as a Self-Supporting Electrode for Supercapacitor and Oxygen Evolution Reaction
Source: Nanomaterials (Basel). 2022 Jun 20;12(12):2121. doi: 10.3390/nano12122121 (PMC9227449; doi:10.3390/nano12122121)
Supplement: Supplementary file 1 [file nanomaterials-12-02121-s001.zip › nanomaterials-1781400-supplementary.pdf]

Supporting Information

Cu_xO-Modified Nanoporous Cu Foil as a Self-Supporting Electrode for Supercapacitor and Oxygen Evolution Reaction

Zhenhan Li, Jianbin Lin, Xin He, Yue Xin, Ping Liang and Chi Zhang *

School of Applied Physics and Materials, Wuyi University, 99 Yingbin Road, Jiangmen 529020, China;
zhenhanli@yeah.net (Z.L.); wylinjianbin@163.com (J.L.); hexinwyu@126.com (X.H.);
xin3231946@163.com (Y.X.); ping_liang@126.com (P.L.)
* Correspondence: chizhang@wyu.edu.cn

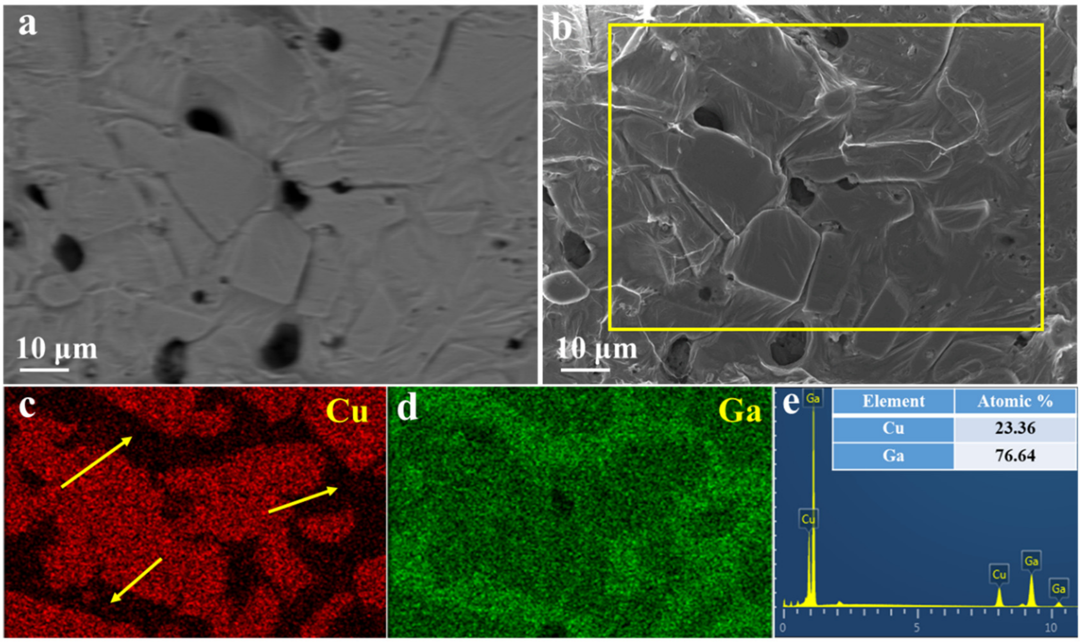

Figure S1. The SEM images in (a) back-scattered mode and (b) InLens mode. The elemental mapping spectra for (c) Cu and (d) Ga. (e) A typical EDX spectrum of the Ga_2Cu layer. The arrows refer to the excessive Ga areas on the surface.

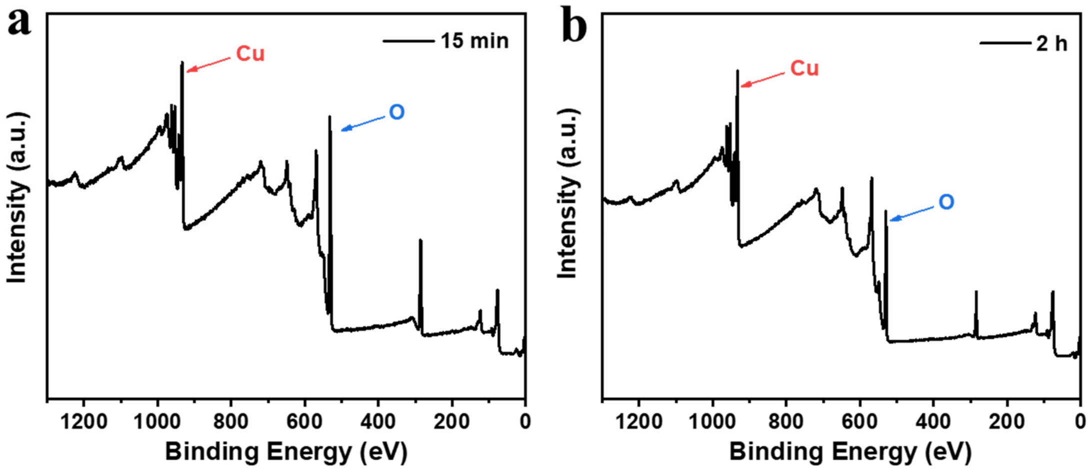

Figure S2. XPS full survey of (a) O-Cu-15m and (b) O-Cu-2h.

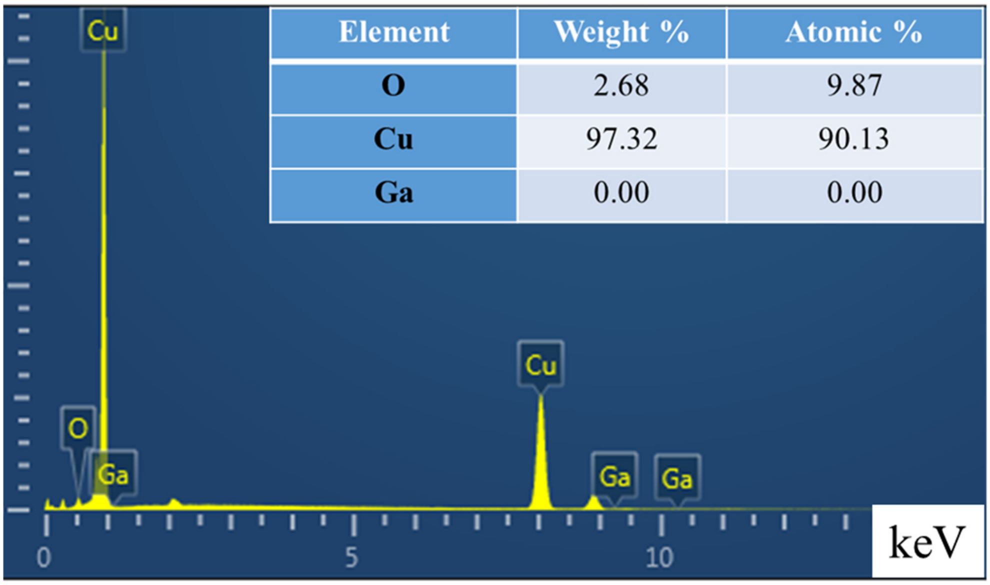

Element	Weight %	Atomic %
O	2.68	9.87
Cu	97.32	90.13
Ga	0.00	0.00

Figure S3. A typical EDX spectrum of the dealloyed Ga_2Cu film corresponding to Figure 2b, indicating that Ga can be fully dealloyed.

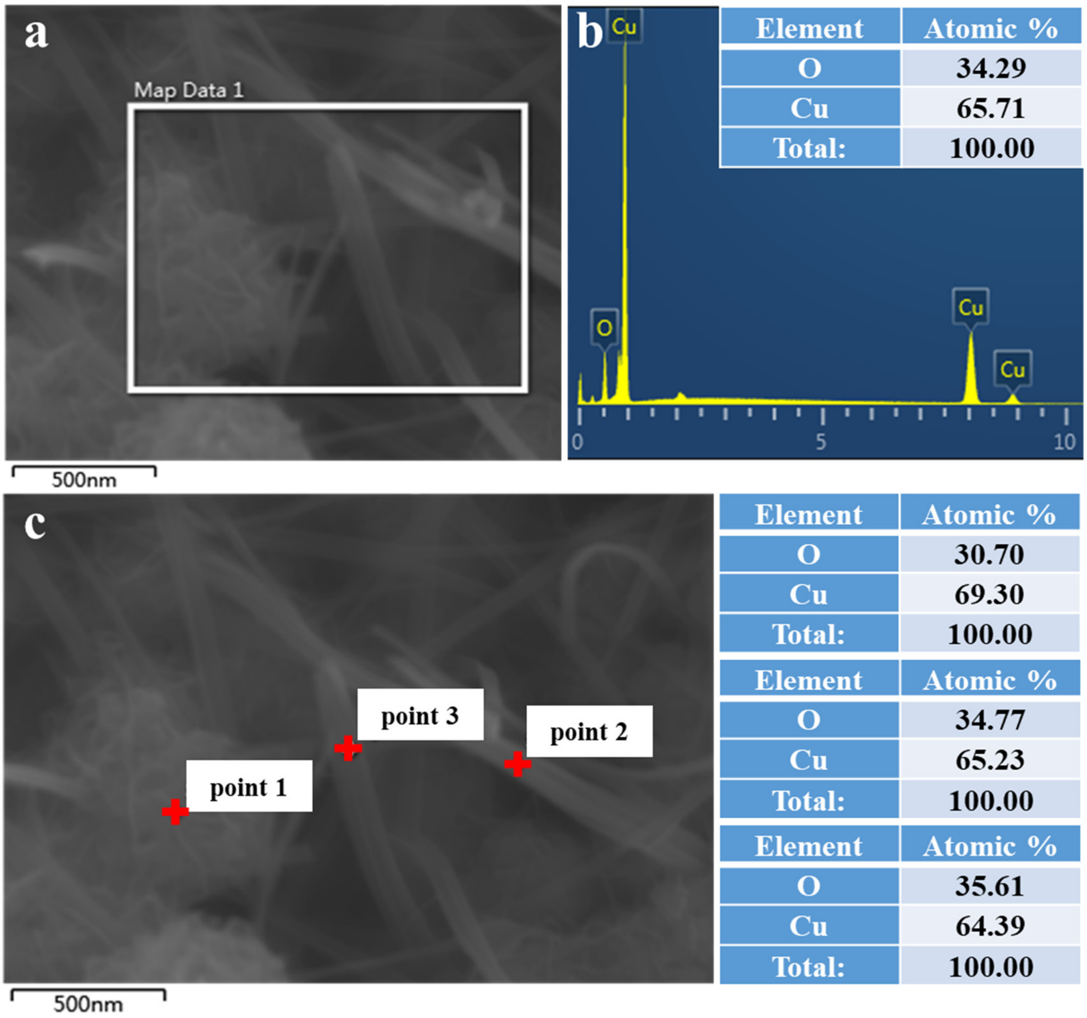

Figure S4. (a) SEM image, (b) the corresponding map sum spectrum of O-Cu-15m, and (c) EDX element atomic concentration from points 1, 2, and 3 (from top to bottom).

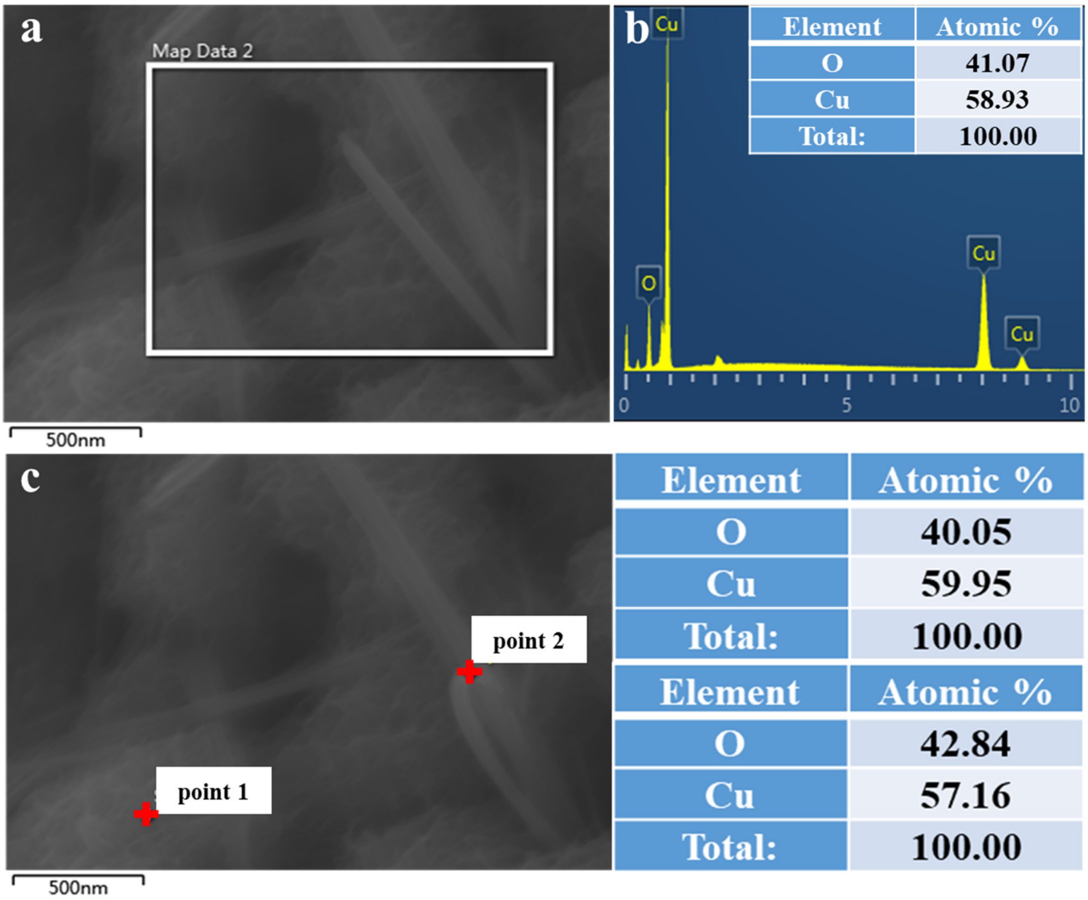

Figure S5. (a) SEM image, (b) the corresponding map sum spectrum of O-Cu-30m, and (c) EDX element atomic concentration from points 1 and 2 (from top to bottom).

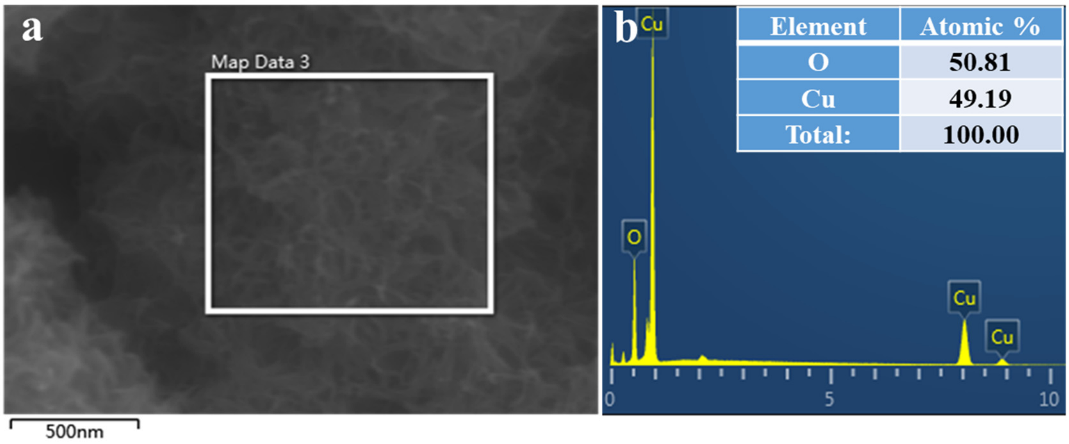

Figure S6. (a) SEM image and (b) the corresponding map sum spectrum of O-Cu-1h.

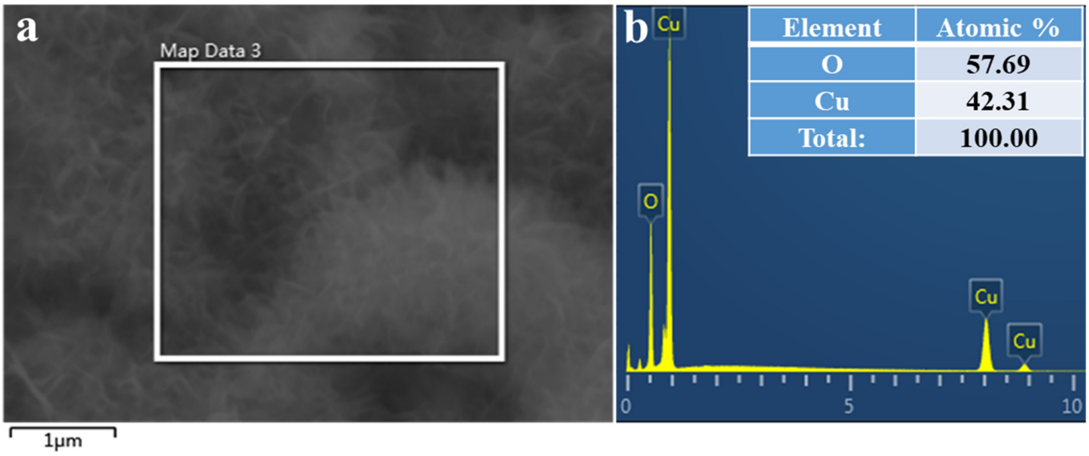

Figure S7. (a) SEM image and (b) the corresponding map sum spectrum of O-Cu-5h.

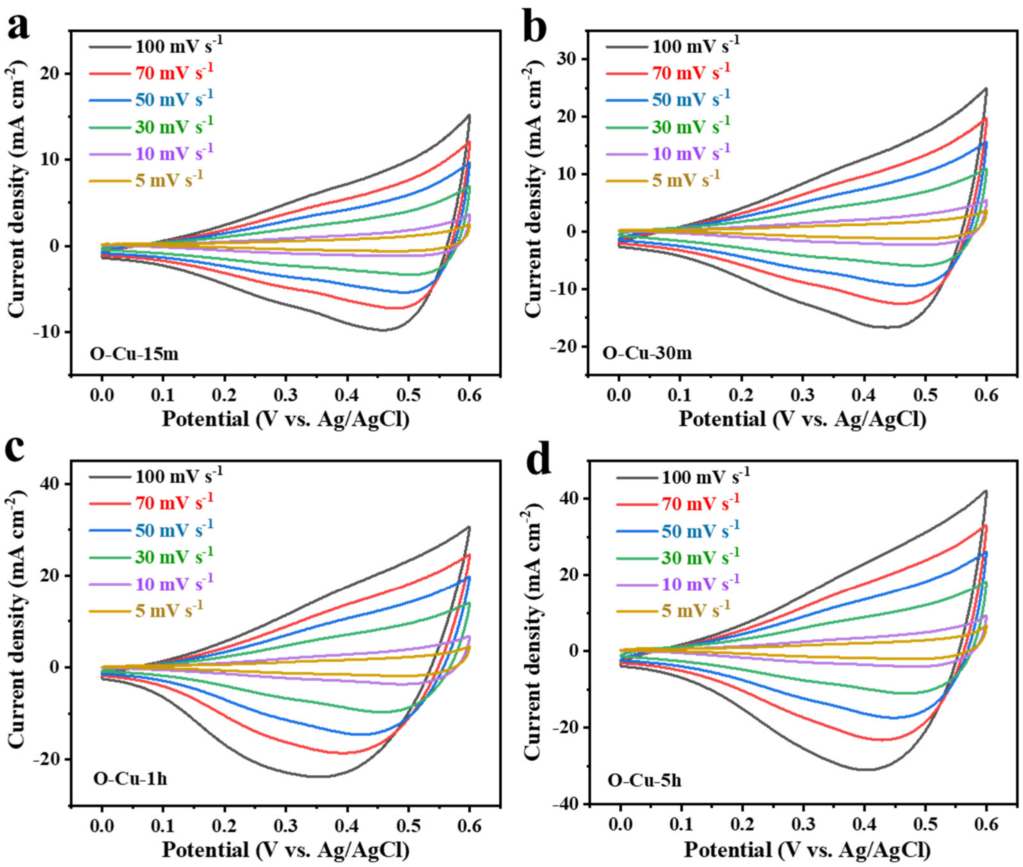

Figure S8. CV curves of O-Cu-15m, O-Cu-30m, O-Cu-1h and O-Cu-5h at various scan rates.

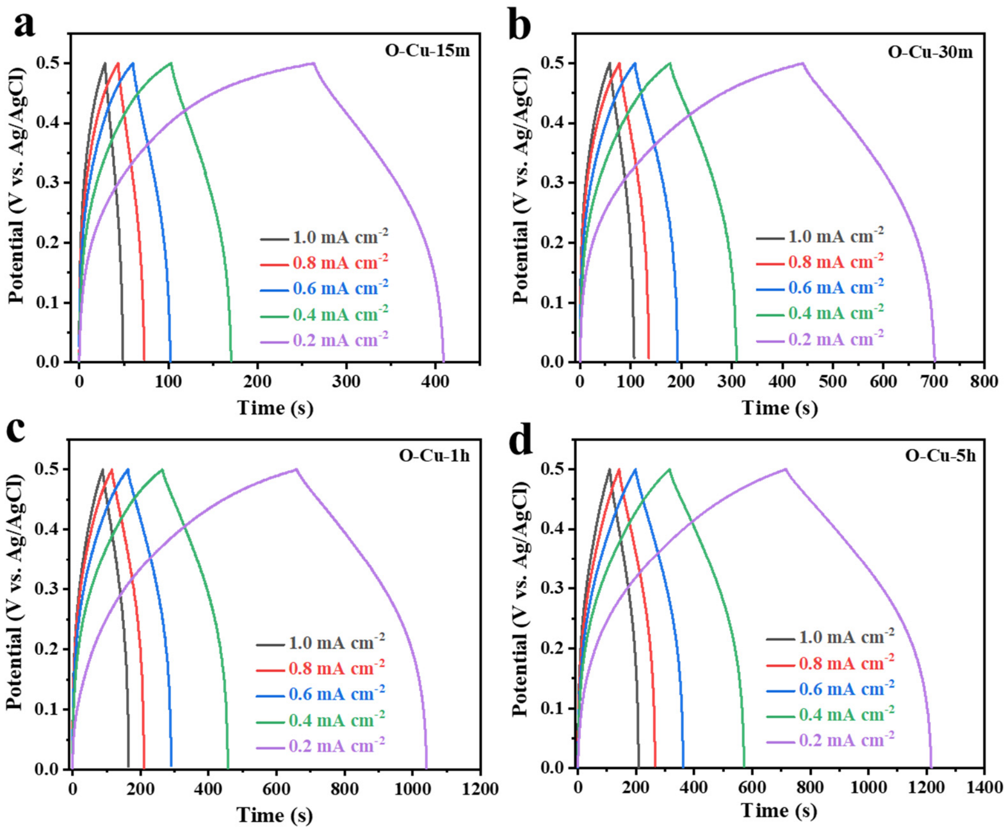

Figure S9. GCD curves of O-Cu-15m, O-Cu-30m, O-Cu-1h and O-Cu-5h at various current densities.

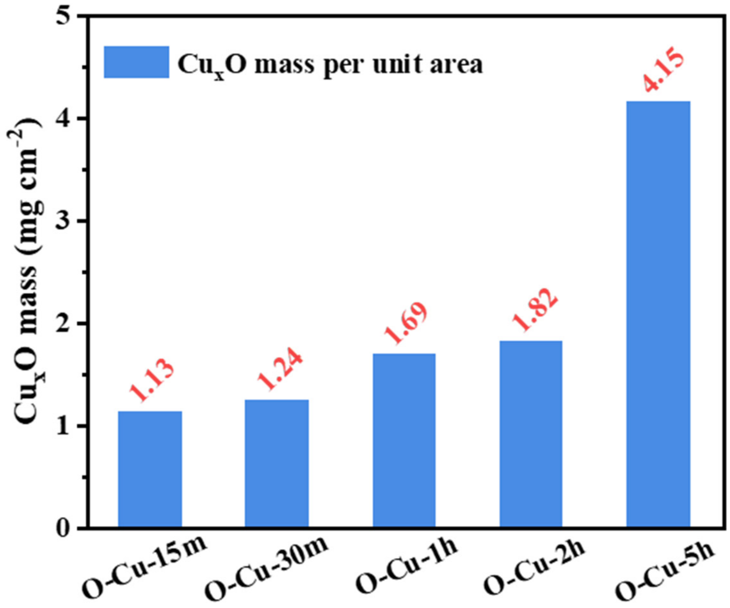

Figure S10. The mass loaing per unit area of Cu_xO on the O-Cu-15m, O-Cu-30m, O-Cu-1h, O-Cu-2h, and O-Cu-5h.

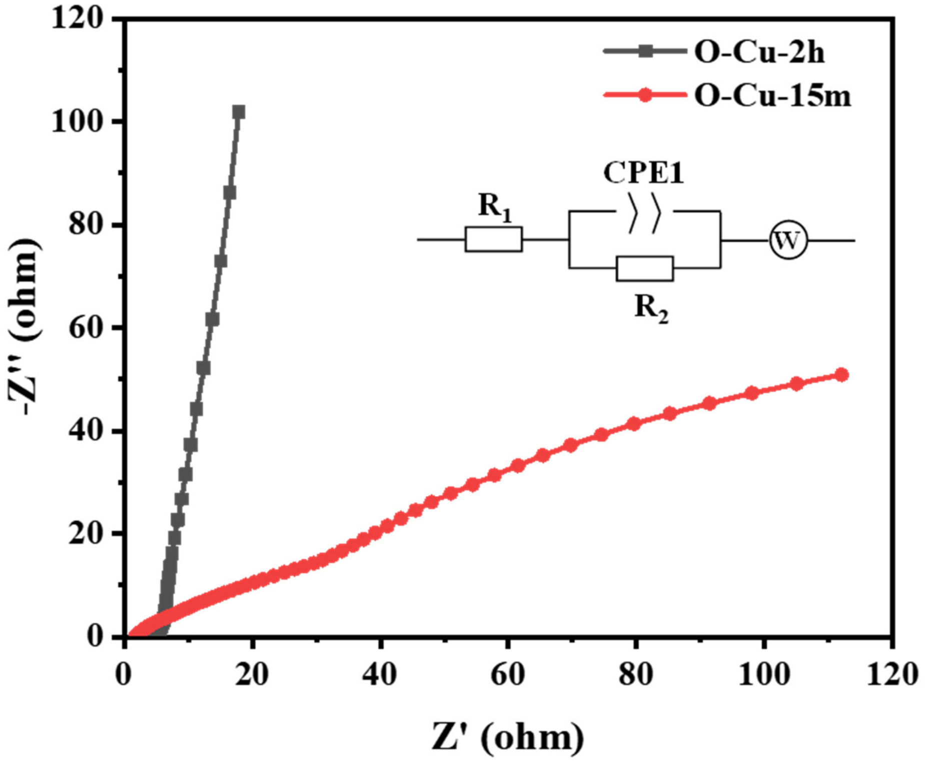

Figure S11. Nyquist impedance plots of O-Cu-2h and O-Cu-15m.

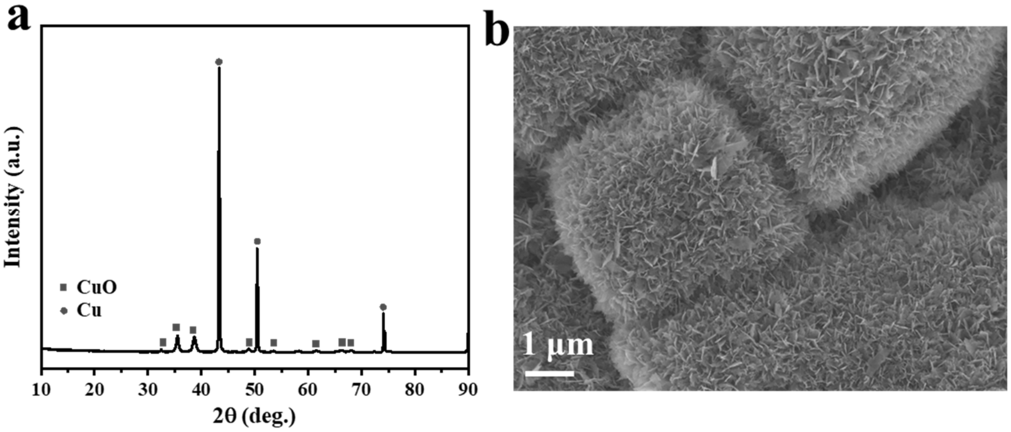

Figure S12. XRD pattern and surface SEM image of O-Cu-2h after 12000 CV cycles at 100 mV s^{-1}.

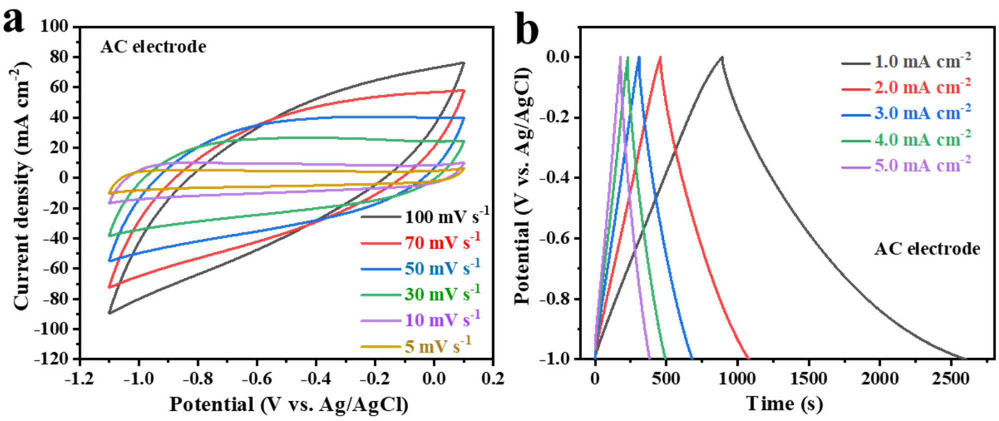

Figure S13. (a) CV curves at various scan rates and (b) GDC curves at current density ranging from 1-5 mA cm⁻².

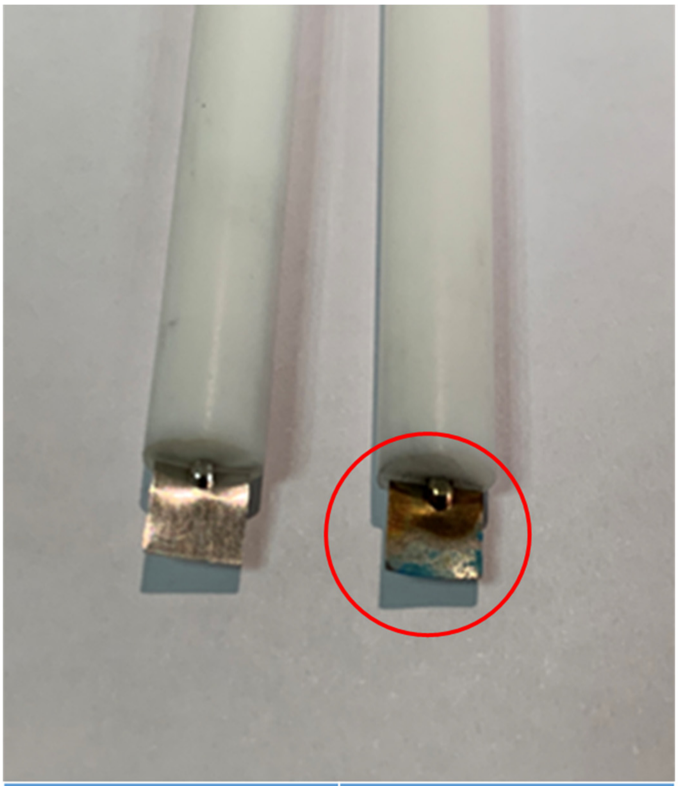

Element	Concentration (mg/L)
Cu	8.23

Figure S14. The comparison photos of Pt electrode before and after i-t test and the ICP result of the electrolyte after i-t test.

Table S1. Comparison of Cu_xO-based electrode for supercapacitors.

Active materials	Current collector	Specific Capacity	Ref.
3D nanostructured Cu_xO	Cu foam	354.6 mF cm^{-2} at 2 mA cm^{-2}	[1]
CuO nanoparticles-biomass-derived carbon	Ni foam	530 F g^{-1} at 1 A g^{-1}	[2]
$Ni(OH)_2$@CuO–CCs	Carbon cloth	2.282 F cm^{-2} at 1 mA cm^{-2}	[3]
$CuCo_2O_4$@CuO	Stainless steel mesh	713 F g^{-1} at 11 mA cm^{-2}	[4]
Cu_xO-C/PANI	Ni foam	1308 F g^{-1} at 1 A g^{-1}	[5]
Porous Cu_xO	Cu foil	156.72 mF cm^{-2} at 1 mA cm^{-2}	[6]
CCCH@NiCo-LDH NWAs@Au–CuO/Cu	Cu fibers	1.97 F cm^{-2} at 7.96 A cm^{-2}	[7]
CuO/3DGN/CC	Carbon cloth	2787 mF cm^{-2} at 6 mA cm^{-2}	[8]
Cu_2O/CuO/RGO	Cu foil	173.4 F g^{-1} at 1 A g^{-1}	[9]
Nanoporous CuO	Ni foam	1.51 F cm^{-2} at 3.5 mA cm^{-2}	[10]
Cu_2O@Cu nanoneedle arrays	Cu foam	862.4 F g^{-1} at 1 mV s^{-1}	[11]
Cu_xO nanoflakes	Cu foil	745 mF cm^{-2} (410.27 F g^{-1}) at 0.2 mA cm^{-2}	This work

Table S2. Comparison of the other asymmetric supercapacitors and this work of energy density and power density.

Materials	Energy density	Power density	Ref.
Cu_xO/CF-60//AC/NF	25 μWh cm^{-2}	3 mW cm^{-2}	[1]
CuO-AC//AC	11.7 W h kg^{-1}	628.73 W kg^{-1}	[2]
$Ni(OH)_2$@CuO–CCs//Fe_2O_3@CuO–CCs	81.12 Wh kg^{-1}	2.88 kW kg^{-1}	[3]
CCO@CuO//rGO	37.43 Wh kg^{-1}	250 W kg^{-1}	[4]
CuxO-C/PANI//ZIF-8NPC	55.1 W h kg^{-1}	862.4 W kg^{-1}	[5]
CCCH@NiCo-LDH NWAs@Au–CuO/Cu//VN/carbon fibers	34.97 Wh kg^{-1}	13.86 kW kg^{-1}	[7]
Nanoporous CuO//AC	19.7 W h kg^{-1}	7 kW kg^{-1}	[10]
Cu_2O@Cu//AC	35.6 W h kg^{-1}	0.9 kW kg^{-1}	[11]
Cu_xO nanoflakes//AC	24.20 Wh kg^{-1}	0.65 kW kg^{-1}	This work

References

1. Li, Y.; Jiang, H.; Yan, X.; Zhu, Y.; Zhang, W.; Zhang, M.; Zhu, W.; Javed, M. S.; Pan, J.; Hussain, S. 3D nanostructured Cu_xO modified copper foam as a binder-free electrode for all-solid-state supercapacitor. *Ceram. Int.* **2021,** *47,* (22), 31138-31148.
2. Zhan, Y.; Bai, J.; Guo, F.; Zhou, H.; Shu, R.; Yu, Y.; Qian, L. Facile synthesis of biomass-derived porous carbons incorporated with CuO nanoparticles as promising electrode materials for high-performance supercapacitor applications. *J. Alloys Compd.* **2021,** *885*.
3. Wu, T.; Xu, L. n.; Sun, H.; Bao, Y.; Yu, H.; Guo, X.; Hu, Q.; Li, J. Hierarchical shell/core electrodes with CuO nanowires based on carbon cloths for high performance asymmetric supercapacitors. *Ceram. Int.* **2021,** *47,* (23), 33758-33765.
4. Kamble, G. P.; Rasal, A. S.; Gaikwad, S. B.; Gurav, V. S.; Chang, J.-Y.; Kolekar, S. S.; Ling, Y.-C.; Ghule, A. V. $CuCo_2O_4$ Nanorods Coated with CuO Nanoneedles for Supercapacitor Applications. *ACS Applied Nano Materials* **2021,** *4,* (11), 12702-12711.
5. Chhetri, K.; Dahal, B.; Mukhiya, T.; Tiwari, A. P.; Muthurasu, A.; Kim, T.; Kim, H.; Kim, H. Y. Integrated hybrid of graphitic carbon-encapsulated Cu_xO on multilayered mesoporous carbon from copper MOFs and polyaniline for asymmetric supercapacitor and oxygen reduction reactions. *Carbon* **2021,** *179,* 89-99.
6. Wang, S.; Jiang, L.; Hu, J.; Wang, Q.; Zhan, S.; Lu, Y. Dual-functional Cu_xO/Cu electrodes for supercapacitors and non-enzymatic glucose sensors fabricated by

femtosecond laser enhanced thermal oxidation. *J. Alloys Compd.* **2020,** *815*, 152105.
7. Guo, Y.; Hong, X.; Wang, Y.; Li, Q.; Meng, J.; Dai, R.; Liu, X.; He, L.; Mai, L. Multicomponent hierarchical Cu-doped NiCo-LDH/CuO double arrays for ultralong-life hybrid fiber supercapacitor. *Adv. Funct. Mater.* **2019,** *29*, (24), 1809004.
8. Li, Y.; Wang, X.; Yang, Q.; Javed, M. S.; Liu, Q.; Xu, W.; Hu, C.; Wei, D. Ultra-fine CuO nanoparticles embedded in three-dimensional graphene network nano-structure for high-performance flexible supercapacitors. *Electrochim. Acta* **2017,** *234*, 63-70.
9. Wang, K.; Dong, X.; Zhao, C.; Qian, X.; Xu, Y. Facile synthesis of Cu_2O/CuO/RGO nanocomposite and its superior cyclability in supercapacitor. *Electrochim. Acta* **2015,** *152*, 433-442.
10. Moosavifard, S. E.; El-Kady, M. F.; Rahmanifar, M. S.; Kaner, R. B.; Mousavi, M. F. Designing 3D highly ordered nanoporous CuO electrodes for high-performance asymmetric supercapacitors. *ACS Appl. Mater. Interfaces* **2015,** *7*, (8), 4851-60.
11. Dong, C.; Wang, Y.; Xu, J.; Cheng, G.; Yang, W.; Kou, T.; Zhang, Z.; Ding, Y. 3D binder-free Cu_2O@Cu nanoneedle arrays for high-performance asymmetric supercapacitors. *J. Mater. Chem. A* **2014,** *2*, (43), 18229-18235.
